# Parents are stressed! Patterns of parent stress across COVID-19

**DOI:** 10.21203/rs.3.rs-66730/v2

**Published:** 2020-12-09

**Authors:** Elizabeth L. Adadms, Danyel Smith, Laura J. Caccavale, Melanie K. Bean

**Affiliations:** 1 Virginia Commonwealth University, Department of Pediatrics, Children’s Hospital of Richmond, Richmond, VA, USA

**Keywords:** viral pandemic_1_, coronavirus_2_, parenting stress_3_, parent coping_4_, stress management_5_, COVID-19_6_

## Abstract

**Background:**

The Coronavirus Disease 2019 (COVID-19) pandemic has caused numerous unexpected challenges for many families, and these long-lasting demands likely contribute to higher stress for parents. The aim of this study was to describe changes in parent stress longitudinally from before (retrospective) to two timepoints during COVID-19. Stressors that influenced parenting and strategies to manage parenting difficulties at each timepoint during COVID-19 are also described.

**Methods:**

Parents (N=433; 95% female) in the US with ≥1 child aged 5–18 years completed an online survey in May 2020 (T1; at the peak of stay-at-home mandates) and in September 2020 (T2; children’s return to school). Surveys included the 10-item Perceived Stress Scale (PSS) and questions on parenting-specific stress, stressors that influenced parenting, and strategies to manage parenting difficulties during COVID-19. Retrospective report of pre-COVID-19 stress was assessed at T1; current stress was assessed at T1 and T2. Repeated measures analysis of variance examined changes in stress over time.

**Results:**

Parent’s stress increased from before COVID-19 to T1 (PSS score: 16.3±5.7 to 22.0±6.4, respectively; p<0.01), and decreased by T2 (19.2±6.0), but remained elevated above pre-COVID-19 values (p<0.01). Most parents (71.1%) reported an increase parenting-specific stress from before COVID-19 to T1, which continued to increase for 55% of parents at T2. Common stressors that impacted parenting during COVID-19 were changes in children’s routines, worry about COVID-19, and online schooling demands. Common strategies parents used to manage parenting difficulties included doing family activities together, keeping in touch with family/friends virtually, and keeping children on daily routines.

**Conclusions:**

Parent stress increased substantially during COVID-19 and has not returned to pre-COVID-19 levels, suggesting the need for enhanced mental health resources and supports. Public health interventions should address parenting-specific stressors and effective strategies for managing parenting difficulties to mitigate their deleterious impact.

## 1.0 Introduction

The Coronavirus Disease 2019 (COVID-19) has swept the globe causing new and unexpected challenges, including severe financial losses, concerns around contracting COVID-19, and mandatory stay-at-home orders disrupting families’ daily routines. These challenges have contributed to a heightened awareness among clinicians (1), researchers (2,3) and public health organizations (4,5) of the potential for substantial increased family stress. Furthermore, given the persistent and repeated demands of this pandemic, many families are likely experiencing chronic stress, which is concerning given the physiological and emotional consequences of chronically elevated stress (6), such as increased risk for cardiovascular disease (7), obesity (8), altered respiratory patterns (9), and depression (10).

Previous infection outbreaks have resulted in profound psychosocial consequences (11,12), and emerging evidence during COVID-19 has shown similar patterns (13,14), particularly for parents (15,16). A recent nationwide poll found that US parents are experiencing higher levels of stress during COVID-19, compared to adults without children, given the added challenges of managing children’s at-home schooling, halts to extracurricular activities, and navigating children’s emotions around uncertainty and change (17). To further this evidence, research is needed to examine how parent stress has changed over the course of this pandemic, and the specific stressors causing parenting difficulties. This is especially important to examine at key timepoints during COVID-19 where parents stress is likely high, including the peak of government closures and stay-at-home orders (approximately May 2020) and upon children’s return to school in Fall (approximately September 2020). These data can be used to better understand the psychological impact of COVID-19 on parents’ mental health and how this pandemic is affecting families over time.

Effective stress management is essential to mitigate the deleterious impact of COVID-19. To manage pandemic-related stress, professional organizations such as the Centers for Disease Control and Prevention (CDC) and World Health Organization (WHO) recommend strategies such as setting a routine, taking time to unwind, connecting with others, and staying informed while limiting the amount of news that causes distress (4,5). However, it is unknown which specific strategies parents are using to effectively manage their stress and parenting difficulties, and the extent to which certain strategies change throughout the course of COVID-19. Data on parents’ use and effectiveness of stress management techniques would be useful to understanding how parents cope over time and to inform initiatives that help improve families’ health and well-being. Targeted public health efforts can then be developed and implemented for prevention and stress management to address parents’ emotional well-being and provide support.

The aim of this paper is to examine changes in parents’ perceived stress, from before to throughout COVID-19. Data were collected at two key timepoints during COVID-19 (May and September 2020). Additionally, at each timepoint, pandemic-related stressors that parents report as having impacted their parenting during COVID-19, and strategies they found effective at managing stress and parenting difficulties are described.

## 2.0 Materials and Methods

### 2.1 Study Design and Participant Sample

A longitudinal, observational study utilizing an online survey was conducted at two separate timepoints in May (T1) and September 2020 (T2). At T1, parents were asked about their general and parenting-specific stress before COVID-19 (retrospective report), as well as currently during COVID-19. Parents were also asked about stressors that currently influenced their parenting and strategies they found effective at managing parenting difficulties during COVID-19. The same survey was administered at T2 where parents only reported on their current perceptions of these constructs.

### 2.2 Procedures

The first online survey administered at T1 occurred during the peak of government-mandated stay-at-home orders between April 30, 2020 and May 23, 2020, approximately four months after the start of the COVID-19 in the US (18) and a few weeks after most states had closed schools (19). Participants were recruited nationwide through: 1) Facebook advertisements targeting parents with lower educational attainment and living in lower-income ZIP codes; and 2) a snowballing technique using emails sent to colleagues across different sectors (e.g., academic, community partners, schools, nonprofit organizations) and postings on social media platforms (e.g., Twitter, parenting forums, and university pages on Facebook). Interested participants were directed to the survey site using Qualtrics, where an informational letter provided details of the study. Passive permission was used to obtain informed consent. Screening questions assessed eligibility, including if participants lived in the US, were ≥18 years of age, and had ≥1 child that was 5–18 years of age. The eligibility criteria for child age was chosen in order to recruit parents of school-aged children affected by school closures. Parents who responded affirmatively to these questions were eligible and prompted to complete the full survey (n=58 parents were not eligible). To ensure that all completed responses were valid (e.g., not bots), the study used Completely Automated Public Turing test to tell Computers and Humans Apart (CAPTCHA) features and the research staff continuously monitored all survey responses. Upon survey completion, each respondent was contacted to provide compensation and ensure the responses were from humans. A total of n=603 parents completed the first survey, and n=19 were excluded due to invalid survey responses from bots (resulting in n=584). Of these, n=433 also completed the second survey at T2 (74% retention). These analyses include the N=433 who completed both surveys. Parents who did *not* complete the second survey were more likely to be Hispanic/Latino (p<0.01) and non-White (p=0.03). There were no other differences for demographic or perceived general stress scores between parents who did vs. did not complete the second survey. The study procedures and passive consent process were approved by the Institutional Review Board at [redacted] University.

### 2.3 Instruments

#### 2.3.1 Perceived stress.

The 10-item Perceived Stress Scale (PSS) was used to measure general stress. At T1, parents completed the PSS when reflecting back on their feelings before COVID-19, and again considering their current feelings during COVID-19. At T2, parents completed the PSS only once when considering their current situation at that time. This reliable and valid scale is the most widely used psychological instrument to evaluate perceived stress (20,21). Items were rated on a 5-point Likert scale ranging *Never* (0) to *Very Often* (4) and summed. Total scores ranged 0 to 40, with higher scores indicating greater stress. Total scores were categorized to describe parents who experienced low stress (0–13), moderate stress (14–26), and high stress (27–40) based on previously established cutoff values (22–25). Parenting-specific stress was assessed at each timepoint: parents reported if their parenting-specific stress *increased*, *decreased*, or *remained the same* since before COVID-19 (at T1) and since May 2020 (at T2).

#### 2.3.2 Parenting difficulty.

Parenting difficulty was assessed at each timepoint using a single-item question asking, “How difficult have the past few weeks been for you to continue parenting in the same as you did prior to COVID-19?” Response options included *not difficult at all*, *somewhat difficult*, *very difficult*, or *extremely difficult*.

#### 2.3.3 Factors influencing parenting.

At each timepoint, parents were provided with a list of 9 possible stressors and asked to select which impacted their parenting during COVID-19. Stressors included a lack of money, lack of food, lack of time due to increased work tasks, lack of time due to increased household tasks, change in daily routines and structure, parents’ worry/anxiety around COVID-19, child’s worry/anxiety around COVID-19, child’s online schooling at home demands, and child’s desire to be with friends. Parents selected all that applied. There was a response option for “other,” where parents were prompted to provide a short answer response. This list of stressors was developed by experts on the research team, given the lack of existing measures assessing COVID-19-specific stressors for parents at the time this study was conducted.

#### 2.3.4 Strategies to manage parenting difficulties.

At each timepoint, parents were provided with a list of nine possible strategies and asked to select which of these they found effective at managing parenting difficulties during COVID-19. Strategies included using extended family for resources, using community resources, keeping in touch with family/friends virtually, keeping their child on a daily routine, doing family activities together, finding ways to effectively manage anxiety, controlling the information they seek on COVID-19, focusing on the big priorities and letting the small tasks go, and taking time for myself. Parents selected all that applied and could also choose “other” to provide an alternative short answer response. This list of strategies was developed by experts on the research team, given the purpose of examining COVID-19-specific strategies, and the lack of COVID-19-specific measures at study onset.

#### 2.3.5 Demographics and COVID-19-specific questions.

At T1 only, parents answered demographic questions including parent age, sex, race, ethnicity, education, marital status, family income, insurance status, and the total number of children and adults in the home. Additional COVID-19-related questions were asked at both timepoints, including family diagnoses, working from home, job layoffs/furloughs, income changes, and unemployment benefits.

### 2.4 Statistical analysis

Means, standard deviations, and percentages were calculated for demographic and COVID-19-related variables as appropriate. A repeated measures analysis of variance model examined patterns of parents’ perceived stress over time, from before to across COVID-19. Pairwise comparisons examined differences during COVID-19 at T1 and T2, relative to before COVID-19 (reference timepoint). A chi square test of independence was used to examine if the distribution of parents with high, moderate, or low stress differed at each timepoint. Furthermore, percentages were calculated for response options pertaining to factors causing parenting difficulties and strategies to manage parenting difficulties at T1 and T2. Thematic analyses were applied to the “other” responses provided, and categories were created. Two researchers (ELA and DS) created categories and independently rated each parent response into one or more categories. Ratings were compared between researchers, and discrepancies were discussed and resolved using a third person (MKB) when needed. Data were analyzed using SAS statistical software, version 9.4 (SAS Institute Inc., Cary, NC, USA), and significance was set at p<0.05.

## 3.0 Results

### 3.1 Demographics and COVID-19 related factors

Parents were mostly female, White, and married or living with a domestic partner; almost half earned a family income of ≤$50,000/year (Table 1). COVID-19 related factors, including family diagnoses, work, and income-related changes, are listed in Table 1. Most (~60%) families experienced a decrease in income from before COVID-19 to T1, while 40% of families experienced a continual decrease through T2. At T2, ~40% of families reported having a family job loss / furlough since the start of COVID-19, and most children (63.1%) were attending virtual school (19.4% in-person; 17.6% hybrid).

### 3.2 Perceived general stress and parenting-specific stress

Average scores on the PSS changed over time (p<0.01). Specifically, scores increased from before COVID-19 to T1 (16.3±5.7 vs. 22.0±6.4; p<0.01), and decreased by T2 (19.2±6.0), but remained significantly higher than to pre-COVID-19 values (p<0.01). Few parents reported high stress before COVID-19 (3.5%), while 22.4% reported high stress at T1 and 12.2% at T2 (Figure 1). Almost three-fourths of parents (71.1%) reported an increase in parenting-specific stress from before COVID-19 to T1, which continued to increase for 55% of parents from T1 to T2. At T1, 85% of parents reported it was at least somewhat difficult to continue parenting in the same way as they did prior to COVID-19, while 45% reported that parenting had become harder at T2, compared to at T1.

### 3.3 Pandemic-related factors impacting parenting during COVID-19

Specific factors that parents felt impacted their parenting during COVID-19 are illustrated in Figure 2, according to parents’ perceived stress category from the PSS at each timepoint. The most common stressor was a change in children’s daily structure and routines (reported by 86% of parents at T1; 69% at T2). A high percentage of parents also reported that their worry and anxiety around COVID-19 (67% at T1; 49% at T2) and demands related to children’s online schooling at home (67% at T1; 60% at T2) impacted their parenting during the pandemic. The overall percentage of parents who reported that a given stressor impacted their parenting tended to decrease from T1 to T2. The greatest decreases in prevalence from T1 to T2 was regarding changes to children’s daily structure and routines (17.6% fewer parents) and parents’ and children’s worry and anxiety around COVID-19 (18.3% and 11.3% fewer parents, respectively). From T1 to T2, an additional 7.2% of parents reported that a lack of time due to increased work tasks and 1.9% reported that a lack of time due to increased household tasks impacted their parenting. There were 103 total unique “other” answers provided (n=60 at T1; n=43 at T2). These responses were categorized (Table 2) with example quotes for each category to provide representation of these answers.

### 3.4 Strategies to manage parenting difficulties

Figure 3 illustrates strategies that parents found effective at managing parenting difficulties during COVID-19, according to perceived stress category at each timepoint. The most common strategies included doing family activities together (reported by 72% of parents at T1; 66% at T2), keeping in touch with family/friends virtually (68% at T1; 56% at T2), and keeping children on a daily routine (53% at T1; 59% at T2). The overall percentage of parents who reported a given strategy was effective at managing their parenting difficulties increased for some strategies and decreased for other strategies over time. Using extended family for resources and keeping their child on a daily routine increased the most (by ~7–8% of parents) from T1 to T2, while using community resources, keeping in touch with family and friends virtually, and controlling the information they seek on COVID-19 decreased the most (by ~8–13% of parents) from T1 to T2. There were N=23 total “other” strategies reported (n=17 at T1; n=6 at T2). Of these, the most common was engaging in hobbies/exercise (n=8; e.g., “We have started taking daily walks outside”; “Escape to Netflix, Hulu”). Other responses included making family changes (n=3; e.g., “Family meals are more often and when we come together after work and school”), adapting their mental and spiritual outlook (n=4; e.g., “accepting the need to be flexible”; “prayer and virtual church”), and cutting back (n=3; e.g., “doing less for my job;” “not listening to the news”).

## 4.0 Discussion

This study showed an increase in general stress from before to during COVID-19 in May 2020; although stress decreased by September 2020, it remained chronically elevated above pre-COVID-19 values. Moreover, parenting-specific stress also increased from before COVID-19 to May 2020 and *continued to increase* for just over half of all parents by September 2020. The majority of parents reported that it was difficult to continue parenting in the same way as they did prior to COVID-19, while almost half reported that parenting had become harder over the course of the pandemic. The most common factors that influenced parenting were changes in children’s daily structure and routines, worry and anxiety around COVID-19, and demands related to children’s online schooling at home. To manage these difficulties, most parents found that doing family activities together, keeping in touch with family/friends virtually, and keeping children on a daily routine were effective. These findings highlight the need to address the stress that families are experiencing and provide adequate resources to manage this stress during COVID-19.

Around the height of government closures and stay-at-home orders (May 2020), one-in-five parents reported high stress, while three-in-four parents reported increased parenting-specific stress. Other studies have found that high stress is a common reaction to this pandemic (26) and that parents are experiencing more stress than non-parents (17). These findings demonstrate that while general stress is decreasing over the course of COVID-19, parenting-specific stress is increasing for many families. Disseminating these findings provides scientific evidence to validate parents’ experiences during this time and highlights the critical need to reduce this burden. To support parents during these unprecedented times, public health messaging should continue to promote healthy ways for coping and provide information on managing stress for parenting-specific challenges. For example, organizations like the CDC, WHO, UNICEF, and others have collaborated to provide open access, online resources for evidence-based strategies in managing parenting stress during COVID-19 (27). Future research is needed to examine if families are using resources such as these, and if they help to mitigate stress. Furthermore, policymakers at multiple levels (e.g., local, state, schools, employers) should consider the potential impact of COVID-19 policy changes on parents’ stress and include thoughtful resources to help mitigate this impact (e.g., providing coordinated strategies for parents to help mitigate the impact of school and childcare closures). There is a dire need for coordinated efforts among policymakers to prioritize these systemic changes to reduce the impact of COVID-19 on parents and families.

The most commonly reported factor that influenced parenting during COVID-19 was a change in children’s daily structure and routines. Most children thrive under predictable routines, which makes them feel safe and secure, contribute to healthy habits, and lead to less problematic behaviors [28]. Structure and routines also benefit parents by helping them feel organized and in control, which increases parenting competence and reduces daily stress (28). In May 2020, about half of families reported that keeping children on a daily routine was effective in managing parenting difficulties, and over time, this percentage increased, suggesting this strategy was working for more families. During this time of unpredictability in a rapidly changing environment, it can be difficult for families to keep a consistent daily routine. Parents who are experiencing high stress and have not yet created a daily routine at home may benefit from creating a schedule together with their children, and parents who have established a daily routine need flexibility to adapt to the ever-changing circumstances. It is important for parents to receive guidance from health care professionals on the importance of creating daily structure and routines, as well as education and support from mental health providers on how to best create adaptable routines with their child.

Routines may include designated time for children’s online schooling, which was another common factor that parent felt greatly impacted their parenting. During COVID-19, many parents have had to take on the additional role of teaching their children from home, amongst other work and household tasks. At a time when almost all schools were closed, two-thirds of parents report that children’s online schooling impacted their parenting, which decreased slightly four months later, when some schools resumed in-person or hybrid instruction. Many parents expressed that limited time due to work and household demands were common stressors, which increased in prevalence over time. Parents are understandably overwhelmed by the many responsibilities and roles they have had to take on; for example, one parent mentioned that, “juggling all responsibilities at the same time instead of getting dedicated time for different responsibilities,” impacted her parenting. In addition to parents having to cope with these changes, children are also coping with the lack of sociability from friends and structure that school and extracurricular activities provide. In fact, just over half of parents at both timepoints reported that children’s desire to be with friends have impacted their parenting. Doing family activities together at home and connecting with family and friends virtually are ways to help with coping, bonding, and providing sociability in an environment that limits social interactions (4,5,27).

In May 2020, another common stressor was managing parents’ worry and anxiety around COVID-19. These findings are similar to another study that found reading/hearing about the severity and contagiousness of COVID-19 was the most commonly experienced stressor among a sample of US adults (29). With an abundance of information available, it is important for families to stay informed, yet to also limit the amount and source of information that may be causing anxiety. Organizations such as the CDC and WHO recommend taking breaks from listening to the news and reading about COVID-19, including posts on social media (4,5). Just under half of parents in this study reported that controlling the information they seek on COVID-19 and finding ways to effectively manage their anxiety were effective in reducing parenting difficulties. Other ways in which parents effectively managed their anxiety and stress included engaging in hobbies and exercise, such as going on family walks, taking time for themselves, and adapting their mental outlook. By September 2020, there was a decrease in the percentage of parents who reporting their worry and anxiety around COVID-19 was impacting their parenting, thus suggesting that perhaps parents are finding ways to effectively manage this anxiety, or that their anxiety has naturally lessened over time.

For some parents, prolonged periods of high stress may result in substantial mental health impacts including greater depression, anxiety, and reduced quality of life (30). These mental health impacts could be temporary during COVID-19; however, high amounts of stress have been associated with maladaptive behavior changes, including substance abuse, eating behavior changes, and excessive alcohol consumption (30), that could persist even after stress dissipates. Given potential for a looming mental health crisis, adequate access to quality mental and behavioral health care is of paramount importance (31). Yet, prior to COVID-19, access to mental health resources did not meet the needs (neither the quality nor quantity) for millions of Americans (32), with particular concern about inadequate access for families with Medicaid. Furthermore, healthcare costs have long been a significant barrier to providing mental health resources for those who are uninsured (33).

During COVID-19, millions of Americans have become unemployed and lost employer-provided insurance, further reducing access to mental health resources; thus, families who are more likely to need these mental health resources may not be able to access them. In response to this, the Society of Behavioral Medicine has issued a policy brief outlining recommendations for increasing access to mental health services to manage parent and family stress (31), the Centers for Medicare & Medicaid Services has expanded access to telehealth services, including the provision of mental health, during COVID-19 (34), and The National Alliance on Mental Illness has provided a COVID-19 resource and information guide for finding free in-person and online mental health support (35). While these initiatives represent initial strategies to address the increased mental health concerns, additional public health and policy response are urgent to prevent an impending mental health crisis (36). Moreover, some insurers have already modified their policies that previously increased telehealth coverage and access due to COVID-19; yet these changes are being made where there is no corresponding reduction in need and the impacts of COVID-19 persist (37, 38).

Study limitations include use of a self-reported questionnaire with retrospective parent reported regarding before-COVID-19. Obtaining baseline measures of parents’ perceived stress was not feasible given the sudden onset of this pandemic. To minimize recall bias, the first survey was administered only a few months after the start of COVID-19, yet responses could be biased in unmeasured ways. This study also used items developed by the research team to assess factors that influenced parenting and effective strategies to manage parenting difficulties. At the time of study onset, measures on COVID-19 specific stressors and parenting strategies did not exist, yet many are now available for future research (39). Response options for these questions may not have encompassed all possible stressors and coping strategies, so to overcome this limitation to some extent, an option of “other” was included where parents could provide an alternative response. These “other” responses represent a small percentage of respondents yet provide rich data and a unique perspective on some of the stressors and coping strategies that parents were using. Other limitations include a sample of mostly mothers with limited racial/ethnic diversity (i.e., mostly White) that is not a nationally representative of all US parents, thus limiting generalizability of these findings; however, this sample did provide a diversity across family income and parent education. Parents who did not complete the second survey tended to be from racial/ethnic minority backgrounds and given these populations often experience greater stress [40], these results could underestimate the stress experienced in more vulnerable, minority populations. Lastly, parental stress could have varied across geographical locations where different policies were enforced at the time of survey completion. Larger, more nationally representative data sets, such as the Stress in America Poll (17), should be referenced for changes in stress across a broader US population and by location.

## 5.0 Conclusion

This study provides timely data regarding the significant increase in parents’ stress over the initial course of COVID-19, as stay-at-home orders were eased, and some children returned to school. Information from this study can be used to advocate for policies that support parents and families, including access to appropriate mental health resources to mitigate the negative impacts for those in need. Given the potential for an impending mental health crisis, adequate access to quality behavioral health services, including remote telehealth options, and workplace policies to accommodate families during this time are necessary for managing the chronic stress experienced by many parents. Specific parenting stressors and strategies parents found effective at managing parenting difficulties can be used to inform stress management initiatives and targeted prevention messaging specific to parenting challenges during the COVID-19 pandemic.

## Figures and Tables

**Figure 1.** Percentage of parents with high, moderate, and low stress based on the Perceived Stress Scale, reported retrospectively for before COVID-19, and concurrently during COVID-19 in May and September 2020. Data were collected in a nationwide sample of US parents with a child 5–18 years of age (N=433).

**Figure 2.** Pandemic-related factors that parents reported as having impacted their parenting during COVID-19 in May and September 2020, illustrated by parent-reported stress levels (low, moderate, or high) at that corresponding timepoint. Data collected in a nationwide sample of US parents with a child 5–18 years of age (N=433). Parents could have selected more than one factor.

**Figure 3.** Parent-reported strategies that were effective for managing parenting difficulties during COVID-19 in May and September 2020, illustrated by reported strecs level (low, moderate, or high) at that corresponding timepoint during the pandemic. Data collected in a nationwide sample of US parents with a child 5–18 years of age (N=433). Parents could choose more than one strategy and selected all that applied.

**Table 1.** Parent and family demographics and COVID-19-related factors in a sample of US parents with a child 5–18 years of age (N=433)

Parent and family demographics		
Age (mean±SD)	40.4±7.4	
Female sex (%)	94.5	
Race (%)		
Asian	3.9	
African American	6.7	
Caucasian/White	84.8	
Other	6.7	
Not Hispanic or Latino (%)	88.5	
Married or living with domestic partner (%)	77.4	
Education (%)		
Some college or less	34.2	
Associates or bachelor’s degree	39.0	
Some graduate training or more	26.8	
Family income ≤$50,000/year (%)	47.8	
Insurance (%)		
Medicaid	35.8	
Private insurance	58.2	
None	6.0	
# children in the home (mean±SD)	2.2±1.2	
# adults in the home (mean±SD)	2.1±0.8	
COVID-19-related factors	May 2020	Sept 2020

Family diagnosis of COVID-19 (%)	10.4	18.7
Parent worked outside the home (%)	51.3	33.0
Decreased family income^a^ (%)	58.9	39.7
Filed for or received unemployment benefits (%)	36.0	19.2

a Decreased family income reported in May 2020 was relative to before COVID-19. Decreased family income in September 2020 was relative to May 2020.

**Table 2.** Parent “other” responses to factors influencing parenting difficulties during the COVID-19 pandemic, categorized by topic

Category	Responses (n)	Example Quotes
Children’s schooling	19	“No ability for my child to have schooling during this time” “Parenting and having my child do their schoolwork” “My stress has increased due to my child physically attending school”

Mental and emotional health	18	“Being in the house has become overwhelming” “My child’s distress over not attending school and activities” “My child’s depression over all the change because of COVID-19”

Parents’ work or graduate school demands	14	“Working from home with children home as well” “Balancing my spouse’s job responsibilities” “A lack of time due to increased school tasks (grad school)”

Parents’ time and resource availability	14	“Worry about possible lack of food and trying to stay healthy” “A lack of time to attend to my own needs (exercise, hobbies, etc.)” “Juggling all responsibilities at the same time instead of getting dedicated time for different responsibilities”

Missing out	8	“Senior year of high school and missing so much…” “Missing organized sports” “My child wanting to play at parks with other kids”

Children’s behavior or special needs	6	“Child’s behavior has gotten worse” “Autism and routine changes” “My child has ADHD and several behavioral issues that have been set backwards. It’s extremely hard to be positive about the regression.”

Medical conditions / death	6	“Death in the family” “My parents being in the hospital with COVID-19 and also me and my husband. My mother being diagnosed with cancer.”

Not seeing extended family	4	“Inability to visit grandparents who live nearby” “Lack of support from extended family members due to social isolation”

Marital conflict	3	“Increased conflict with spouse” “Marital strain”

*Note*: These responses were provided when parents selected “other” in response to survey questions that asked about factors influencing parenting difficulties during COVID-19. A single response could fall into more than one category. N=103 “other” responses.

## References

[1] Pfefferbaum B, North CS. Mental health and the Covid-19 pandemic. New Engl J Med. 2020 10.1056/NEJMp2008017 32283003

[2] McGuire JF. Stressed about COVID-19? Here’s what can help. Johns Hopkins Medicine 2020 Available at: https://www.hopkinsmedicine.org/health/conditions-and-diseases/coronavirus/stressed-about-covid19-heres-what-can-help. Accessibility verified August 25, 2020.

[3] Yale Health. COVID-19 managing stress: stress, coping and living a healthy life at home. 2020 Available at: https://yalehealth.yale.edu/covid-19-managing-stress. Accessibility verified August 25, 2020.

[4] World Health Organization. Looking after our mental health. #HealthyAtHome – Mental health. Available at: https://www.who.int/campaigns/connecting-the-world-to-combat-coronavirus/healthyathome/healthyathome---mental-health. Accessibility verified August 25, 2020.

[5] Centers for Disease Control and Prevention. Coping with stress. Coronavirus disease 2019 (COVID-19). National Center for Immunization and Respiratory Diseases, Division of Viral Diseases. 2020 Available at: https://www.cdc.gov/coronavirus/2019-ncov/daily-life-coping/managing-stress-anxiety.html. Accessibility verified August 25, 2020.

[6] Thoits PA. Stress and health: major findings and policy implications. J Health Soc Behav. 2010; 51(1): S41–S53. 20943582 10.1177/0022146510383499

[7] Cohen BE, Edmondson D, Kronish IM. State of the art review: depression, stress, anxiety, and cardiovascular disease. Am J Hypertens. 2015; 28(11): 1295–1302. 25911639 10.1093/ajh/hpv047 PMC4612342

[8] Tomiyama AJ. Stress and obesity. Ann Rev Psychol. 2019; 70: 703–718. 29927688 10.1146/annurev-psych-010418-102936

[9] Taelman J, Vandeput S, Spaepen A, Van Huffel S. Influence of mental stress on heart rate and heart rate variability. In: Vander Sloten J, Verdonck P, Nyssen M, Haueisen J, eds. 4th European Conference of the International Federation for Medical and Biological Engineering Heidelberg Berlin: Springer; 2008: 1366–1369.

[10] Yang L, Zhao Y, Want Y, The effects of psychological stress on depression. Curr Neuropharmacol. 2015; 13(4): 494–504. 26412069 10.2174/1570159X1304150831150507 PMC4790405

[11] Rubin GJ, Potts HWW, Michie S. The impact of communications about swine flu (influenza A H1N1v) on public responses to the outbreak: Results from 36 national telephone surveys in the UK. Health Technol Assess. Health Technol Assess. 2010; 14: 183–266. 20630124 10.3310/hta14340-03

[12] Van Bortel T. Psychosocial effects of an Ebola outbreak at individual, community and international levels. B World Health Organ. 2016; 94(3): 210–214. 10.2471/BLT.15.158543 PMC4773931 26966332

[13] Wang C, Pan R, Wan X, Immediate psychological responses and associated factors during the initial stage of the 2019 coronavirus disease (COVID-19) epidemic among the general population in China. Int J Environ Res Public Health. 2020; 17(5): 1729. 10.3390/ijerph17051729 PMC7084952 32155789

[14] Palsson OS, Ballou S, Gray S. The U.S. national pandemic emotional impact report. 2020 Available at: https://www.pandemicimpactreport.com/report/PalssonBallouGray_2020PandemicImpactReport.pdf. Accessibility verified October 12, 2020. 10.1016/j.bbih.2020.100161 PMC7568491 33103127

[15] Calarco J, Anderson E, Meanwell E, Knopf A. “Let’s not pretend it’s fun”: How COVID-19-related school and childcare closures are damaging to mothers’ well-being. SocArXiv. 2020 10.31235/osf.io/jyvk4

[16] Achterberg A, Dobbelaar S, Boer O, Crone EA. Home lockdown: bloom or boom? Perceived stress as mediator for longitudinal effects of the COVID-19 lockdown on wellbeing of parents and children. Available at: https://www.google.com/url?sa=t&rct=j&q=&esrc=s&source=web&cd=&ved=2ahUKEwijzIGg1q_sAhUNhXIEHf0-ANIQFjABegQIAhAC&url=https%3A%2F%2Fpsyarxiv.com%2Fpj3sg%2Fdownload&usg=AOvVaw0xyI1qcBqxWwVaZSct-LXt. Accessibility verified October 12, 2020. 10.1038/s41598-021-81720-8 PMC7859207 33536464

[17] American Psychological Association. Stress in the time of COVID-19. Volume one. 2020 Available at: https://www.apa.org/news/press/releases/stress/2020/report. Accessibility verified August 25, 2020.

[18] Holshue ML, DeBolt C, Lindquist S, First case of 2019 novel coronavirus in the United States. New Engl J Med. 2020; 382: 929–936. 32004427 10.1056/NEJMoa2001191 PMC7092802

[19] Education Week. Coronavirus and school closures. 2020 Available at: https://www.edweek.org/ew/section/multimedia/map-coronavirus-and-school-closures.html. Accessibility verified August 25, 2020.

[20] Cohen S, Kamarck T, Mermelstein R. A global measure of perceived stress. J Health Soc Behav. 1983; 4: 386–396. 6668417

[21] Lee EH. Review of the psychometric evidence of the perceived stress scale. Asian Nurs Res. 2012; 6(4): 121–127. 10.1016/j.anr.2012.08.004 25031113

[22] Islam MM, Ekuni D, Toyama N, Associations between sleep quality and duration and periodontal disease among university students: a cross-sectional study. Int J of Environ Res Public Health. 2020; 17(9): 3034. 10.3390/ijerph17093034 PMC7246449 32349308

[23] Kim D, Sung H, Lee J, Cho E. The relationship between perceived stress scale and carotid intima-media thickness using ultrasonography in university students. Korean J Clin Lab Sci. 2017; 49: 308–315.

[24] Thangaraj S, D’Souza L. Prevalence of stress levels among first year medical undergraduate students. Int J Interdiscip Multidiscip Stud. 2014; 1(5): 176–181.

[25] Swaminathan A, Viswanathan S, Gnanadurai T, Perceived stress and sources of stress among first-year medical undergraduate students in a private medical college – Tamil Nadu. Natl J Physiol Pharm Pharmacol. 2015; 6(1): 9–14.

[26] Rajkumar RP. COVID-19 and mental health: A review of the existing literature. Asian J Psychiatr. 2020; 52: 102066. 32302935 10.1016/j.ajp.2020.102066 PMC7151415

[27] Parenting for Lifelong Health. COVID-19: 24/7 Parenting. 2020. Available at: https://www.covid19parenting.com. Accessibility verified August 25, 2020.

[28] Fiese BH, Tomcho TJ, Douglas M, Josephs K, Poltrock S, Baker T. A review of 50 years of research on naturally occurring family routines and rituals: cause for celebration? J Fam Psychol. 2002; 16(4): 381–390. 12561283 10.1037//0893-3200.16.4.381

[29] Park CL, Russell BS, Fendrich M, Finkelstein-Fox L, Hutchinson M, Becker J. Americans’ COVID-19 stress, coping, and adherence to CDC guidelines. J Gen Intern Med. 2020; 1–8. 10.1007/s11606-020-05898-9 PMC7259430 32472486

[30] Schneiderman N, Ironson G, Siegel SD. STRESS AND HEALTH: Psychological, behavioral, and biological determinants. Annu Rev Clin Psychol. 2005; 1: 607–628. 17716101 10.1146/annurev.clinpsy.1.102803.144141 PMC2568977

[31] Bean MK, Adams EL, Buscemi J, Increase access to mental health services due to COVID-19-related parent and family stress. Society of Behavioral Medicine Policy Brief. 2020 [under review]. 10.1093/tbm/ibab063 PMC8195166 34048585

[32] Mental Health in America. 2020 State of Mental Health in America Report. 2020 Available at: https://mhanational.org/sites/default/files/State%20of%20Mental%20Health%20in%20America%20-%202020.pdf. Accessibility verified August 25, 2020.

[33] Rowan K, McAlpine D, Blewett L. Access to cost barriers to mental health care by insurance status, 1999 to 2010. Health Aff (Millwood). 2013; 32(10): 1723–1730. 24101061 10.1377/hlthaff.2013.0133 PMC4236908

[34] Centers for Medicare & Medicaid Services. COVID-19 emergency declaration blanket waivers for health care providers. 2020 Available at: https://www.cms.gov/files/document/summary-covid-19-emergency-declaration-waivers.pdf. Accessibility verified August 25, 2020.

[35] National Alliance on Mental Illness. COVID-19 Resource and Information Guide. 2020 Available at: https://www.nami.org/Support-Education/NAMI-HelpLine/COVID-19-Information-and-Resources/COVID-19-Guide.pdf. Accessibility verified August 25, 2020.

[36] Wan W. The coronavirus pandemic is pushing America into a mental health crisis. The Washington Post. 2020 Available at: https://www.washingtonpost.com/health/2020/05/04/mental-health-coronavirus/. Accessibility verified August 25, 2020.

[37] United Healthcare. Talk to a doctor from home using telehealth services. https://www.uhc.com/health-and-wellness/health-topics/covid-19/telehealth-options. Accessibility verified October 16, 2020.

[38] Anthem. Information from Anthem for care providers about COVID-19. https://providernews.anthem.com/georgia/article/information-from-anthem-for-care-providers-about-covid-19-4. Accessibility verified October 16, 2020.

[39] U.S. Department of Health and Human Services. NIH public health emergency and disaster research response (DR2). NIH repository of COVID-19 research tools. https://dr2.nlm.nih.gov. Accessibility verified October 24, 2020.

[40] Nomaguchi K, House AN. Racial-ethnic disparities in maternal parenting stress: the role of structural disadvantages and parenting values. J Health Soc Behav. 2013; 54(3): 386–404. 24026535 10.1177/0022146513498511 PMC3836435

